# End of life distress as an economic issue: The case for psychedelic-assisted therapy

**DOI:** 10.1017/S1478951526101722

**Published:** 2026-01-28

**Authors:** Michel Dorval, Virginie Audet-Croteau, Sue-Ling Chang, Florence Moureaux, Jason Robert Guertin

**Affiliations:** 1 Faculty of Pharmacy, Université Laval, Québec City, QC, Canada; 2 Department of Oncology, CHU de Québec-Université Laval Research Center, Québec City, QC, Canada; 3 Institut de soins palliatifs et de fin de vie Michel-Sarrazin — Université Laval, Québec City, QC, Canada; 4 Réseau québécois de recherche en soins palliatifs et de fin de vie (RQSPAL), Québec, QC, Canada; 5 Faculty of Medicine, Université Laval, Québec City, QC, Canada; 6 Patient-Partner; 7 Department of Population Health and Optimal Health Practices, CHU de Québec-Université Laval Research Center, Québec City, QC, Canada^‡^

The term ‘end of life’ is often used to refer to the final days or weeks preceding death. While this framing is deeply embedded in public discourse and clinical practice, it only partially reflects the lived reality of many people with life-threatening illnesses, including some metastatic cancers and neurodegenerative disorders. For these individuals, the end of life is not a discrete moment but a prolonged trajectory, sometimes spanning several years, marked by uncertainty, ongoing treatment, and repeated confrontation with the perspective of death. This trajectory is frequently accompanied by persistent existential distress, which does not only affect quality of life but also shapes patterns of health care utilization. Anxiety, depression, fear of disease progression, and loss of meaning influence how patients engage with health care systems across the illness trajectory. Existential suffering can thus be considered a determinant of costs which is often not considered in conventional economic analyses of care for people living with life-threatening illnesses (May et al. 2014).

In publicly funded health care systems, expenditures associated with life-threatening illness are not solely attributable to disease-specific treatments but also to the management of psychological issues occurring alongside disease progression. Studies in palliative and end-of-life care have shown that unmanaged distress is associated with higher use of acute hospital services and a greater likelihood of intensive interventions near death, resulting in higher overall costs (Wright et al. 2008; Zhang et al. 2009). These episodes mobilize significant clinical and organizational resources without necessarily altering the overall illness trajectory.

Psychedelic-assisted therapy (PAT), particularly with psilocybin, is an emerging palliative care intervention with an atypical economic profile. Unlike approaches that rely on chronic or repeated treatments, PAT is typically delivered as a time-limited, structured, and intensive intervention, combining preparatory sessions, 1 or 2 dosing sessions, and psychotherapeutic integration within a defined therapeutic framework. Although it requires a concentrated initial investment of clinical and organizational resources, its objective is a durable shift in how patients experience and relate to life-threatening illness. Available clinical evidence indicates that this approach can lead to rapid and sustained reductions in anxiety and depression among patients with advanced disease, with effects persisting for months after treatment (Griffiths et al. 2016; Ross et al. 2016; Agin-Liebes et al. 2020).

From a health system standpoint, the potential value of PAT lies less in its immediate cost than in its capacity to influence costly care trajectories. Psychological stabilization may reduce the frequency of emergency department visits. It may also support deprescribing, thereby limiting polypharmacy and associated adverse effects. In addition, greater existential clarity may facilitate earlier and more stable alignment of care with patients’ values, potentially reducing the use of intensive interventions late in the illness trajectory whose benefits are often limited (Boston et al. 2011; Bélanger et al. 2025). The potential economic impact of PAT extends beyond patients themselves. Family members and caregivers are often involved over prolonged periods and bear substantial emotional and organizational burdens. Caregiver exhaustion, work absenteeism, and increased use of mental health and social services represent other significant indirect costs.

Economic evaluations of PAT will certainly be challenging. In practice, economic evaluations of health technologies assess the average incremental costs in function of the average incremental effectiveness of patients assigned to 2 different interventions. In the context of existential distress, however, there is no widely accepted or standardized reference intervention against which PAT can be meaningfully compared. Current approaches including pharmacotherapy, psychotherapy, and spiritual support are most often delivered in an individualized manner and have demonstrated modest or inconsistent effects in durably alleviating existential distress among patients with life-threatening illnesses. This absence of a clearly defined effective comparator complicates the application of conventional incremental cost-effectiveness frameworks to interventions specifically targeting existential suffering. Moreover, PAT is currently being administered to small and heterogeneous patient populations raising the risk of highly variable downstream costs (savings) and effectiveness. Furthermore, and as previously mentioned, patients’ existential distress is also likely to impact their caregivers and families which can also lead to added costs. Unfortunately, although systems are in place throughout Canada and abroad to capture health system-specific costs, capturing patient and family-borne costs are quite limited. As financial benefits resulting from PAT will tend to occur outside the health care system, omitting these from the economic evaluation will disadvantage PAT’s economic profile. Such challenges have been documented in economic evaluations of palliative care interventions that act on global trajectories rather than isolated clinical events (Gwyther et al. 2026).

Moreover, experience under Canada’s Special Access Program illustrates how case-by-case authorization for psilocybin requires extensive clinical justification, administrative coordination, and institutional oversight, all of which consume substantial professional and organizational resources (Garel et al. 2025). Without a transition toward a more streamlined regulatory model, the costs associated with access to psilocybin may outweigh potential savings from reduced health care utilization, limiting the sustainability of PAT.

In Québec, which currently reports the highest rates of medical assistance in dying (MAiD) worldwide (Commission sur les soins de fin de vie 2025), these economic considerations are particularly relevant. Requests for MAiD often arise in contexts of profound and multifaceted distress, in which existential dimensions coexist with physical symptoms (Downar et al. 2023). Such distress may be preceded by high and costly health care utilization, including repeated consultations, complex pharmacotherapy, and sustained involvement of interdisciplinary teams. Without questioning the legitimacy of MAiD or patients’ rights to access it, this observation invites reflection on whether greater attention to interventions that durably alleviate existential distress earlier in the illness trajectory might contribute to more stable decision-making and, potentially, to more efficient use of health care resources.

Recognizing existential suffering as a legitimate economic issue invites a re-examination of how end-of-life interventions are assessed. This does not require opposing humanistic values to economic responsibility. Rather, it acknowledges that how a society responds to suffering directly shapes the organization, efficiency, and sustainability of its health care system. As growing numbers of people live for extended periods with life-threatening illnesses, failing to address existential distress as a determinant of care trajectories may perpetuate structural inefficiencies at considerable human and economic cost.

## References

[ref1] Agin-Liebes G, Malone T, Yalch MM, et al. (2020) Long-term follow-up of psilocybin-assisted psychotherapy for psychiatric and existential distress in patients with life-threatening cancer. *Journal of Psychopharmacology* 34(2), 155–166. doi:10.1177/0269881119897615. 31916890

[ref2] Bélanger A, Chang SL, Stephan JF, et al. (2025) Supporting meaningful choices: a decision aid for individuals facing existential distress and considering psilocybin-assisted therapy. *Healthcare* 13(18), 2290. doi:10.3390/healthcare13182290. PMC12469295 41008422

[ref3] Boston P, Bruce A and Schreiber R (2011) Existential suffering in the palliative care setting: an integrated literature review. *Journal of Pain and Symptom Management* 41(3), 604–618. doi:10.1016/j.jpainsymman.2010.05.010 21145202

[ref4] Commission sur les soins de fin de vie (2025) *Rapport Annuel D’activités*. Québec, Canada: Gouvernement du Québec.

[ref5] Downar J, MacDonald S and Buchman S (2023) What drives requests for MAiD? *CMAJ* 195(40), E1385–E1387. doi:10.1503/cmaj.230259 37844931 PMC10581715

[ref6] Garel N, Plourde L, Greenway KT, et al. (2025) The promise and challenges of psychedelic-assisted therapy: lessons from Canada’s special access program. *Nature Mental Health* 3, 756–759. doi:10.1038/s44220-025-00446-y

[ref7] Griffiths RR, Johnson MW, Carducci MA, et al. (2016) Psilocybin produces substantial and sustained decreases in depression and anxiety in patients with life-threatening cancer: a randomized double-blind trial. *Journal of Psychopharmacology* 30(12), 1181–1197. doi:10.1177/0269881116675513. 27909165 PMC5367557

[ref8] Gwyther L, Bates MJ, Tran B, et al. (2026) Economic benefits of investment in palliative care: an appraisal of current evidence and call to action. *Journal of Pain and Symptom Management* 71(1), e69–e81. doi:10.1016/j.jpainsymman.2025.09.020. 40992639

[ref9] May P, Normand C and Morrison RS (2014) Economic impact of palliative care interventions. *BMJ Supportive and Palliative Care* 4(3), 1–7. doi:10.1089/jpm.2013.0594

[ref10] Ross S, Bossis A, Guss J, et al. (2016) Rapid and sustained symptom reduction following psilocybin treatment for anxiety and depression in patients with life-threatening cancer. *Journal of Psychopharmacology* 30(12), 1165–1180. doi:10.1177/0269881116675512. 27909164 PMC5367551

[ref11] Wright AA, Zhang B, Ray A, et al. (2008) Associations between end-of-life discussions, patient mental health, medical care near death, and caregiver bereavement adjustment. *JAMA* 300(14), 1665–1673. doi:10.1001/jama.300.14.1665. 18840840 PMC2853806

[ref12] Zhang B, Wright AA, Huskamp HA, et al. (2009) Health care costs in the last week of life. *Archives of Internal Medicine* 169(5), 480–488. doi:10.1001/archinternmed.2008.587. 19273778 PMC2862687

