# End of life distress as an economic issue: The case for psychedelic-assisted therapy – ERRATUM

**DOI:** 10.1017/S1478951526101771

**Published:** 2026-03-10

**Authors:** Michel Dorval, Virginie Audet-Croteau, Sue-Ling Chang, Florence Moureaux, Jason Robert Guertin

**Affiliations:** 1 Faculty of Pharmacy, Université Laval, Québec City, QC, Canada; 2 Department of Oncology, CHU de Québec-Université Laval Research Center, Québec City, QC, Canada; 3 Institut de soins palliatifs et de fin de vie Michel-Sarrazin — Université Laval, Québec City, QC, Canada; 4 Réseau québécois de recherche en soins palliatifs et de fin de vie (RQSPAL), Québec, QC, Canada; 5 Faculty of Medicine, Université Laval, Québec City, QC, Canada; 6 Patient-Partner; 7 Department of Population Health and Optimal Health Practices, CHU de Québec-Université Laval Research Center, Québec City, QC, Canada

The Publisher apologises for three errors in the author affiliations in the article. These have been corrected in the published article, as follows:

^1^Faculty of Pharmacy, Université Laval, Québec City, QC, Canada; ^2^Department of Oncology, CHU de Québec-Université Laval Research Center, Québec City, QC, Canada; ^3^Institut de soins palliatifs et de fin de vie Michel-Sarrazin — Université Laval, Québec City, QC, Canada; ^4^Réseau québécois de recherche en soins palliatifs et de fin de vie (RQSPAL), Québec, QC, Canada; ^5^Faculty of Medicine, Université Laval, Québec City, QC, Canada; ^6^Patient-Partner and ^7^Department of Population Health and Optimal Health Practices, CHU de Québec-Université Laval Research Center, Québec City, QC, Canada.
